# Improvement in *Neisseria gonorrhoeae* culture rates by bedside inoculation and incubation at a clinic for sexually transmitted infections

**DOI:** 10.1186/s12941-023-00576-0

**Published:** 2023-04-18

**Authors:** LM Brendefur Corwin, P Campbell, K Jakobsen, F Müller, X Lai, M Unemo, TM Leegaard, J Vildershøj Bjørnholt, AO Olsen

**Affiliations:** 1 Department of Microbiology, Oslo University Hospital, Oslo, Norway; 2 Institute of Clinical Medicine, University of Oslo, Oslo, Norway; 3 Department of Microbiology and Infection Control, Akershus University Hospital, Lørenskog, Norway; 4 National Advisory Unit for Sexually Transmitted Infections, Oslo University Hospital, Oslo, Norway; 5 Department of Biostatistics, Institute of Basic Medical Sciences, University of Oslo, Oslo, Norway; 6 WHO Collaborating Centre for Gonorrhoea and other STIs, National Reference Laboratory for STIs, Department of Laboratory Medicine, Faculty of Medicine and Health, Örebro University, Örebro, Sweden; 7 Institute for Global Health, University College London (UCL), London, UK; 8 Section for Respiratory, Blood-borne and Sexually Transmitted Infections, Department of Infection Control and Vaccines, Norwegian Institute of Public Health, Oslo, Norway; 9 Department of Community Medicine and Global Health, Institute of Health and Society, University of Oslo, Oslo, Norway

**Keywords:** *Neisseria gonorrhoeae*, Gonorrhoea, Culture, Antibiotic resistance

## Abstract

**Background:**

Culture of *Neisseria gonorrhoeae* is essential for surveillance of complete antimicrobial susceptibility profiles. In 2014, the culture success rate of *N. gonorrhoeae* from samples taken at the clinic for sexually transmitted infections (STI clinic), Oslo University Hospital, Norway, was only 20%. The present study aimed to improve gonococcal culture rates using bedside inoculation of patient samples on gonococcal agar plates and incubation at the STI clinic.

**Methods:**

This prospective quality improvement study was conducted by the STI clinic and the Department of Microbiology at Oslo University Hospital from May 2016 - October 2017. When culture of *N. gonorrhoeae* was clinically indicated, we introduced a parallel ‘bedside culture’ at the STI clinic and compared results with the standard culture at the microbiology department. Samples were taken from urethra, anorectum, pharynx and cervix. Culture rates were compared across symptomatic and asymptomatic anatomical sites.

**Results:**

From 596 gonococcal-positive PCR samples, bedside culture had a significantly higher success rate of 57% compared to 41% with standard culture (p < 0.05). Overall, culture rate from symptomatic sites was 91% *v.* 45% from asymptomatic sites. The culture rates from different anatomical sites were as follows: urethra 93%, anorectum 64%, pharynx 28% and cervix 70%. Bedside culture significantly (p < 0.05) improved the culture rates for symptomatic urethral and asymptomatic pharyngeal samples.

**Conclusions:**

Where feasible, bedside inoculation on gonococcal agar plates and incubation of samples from patients with gonorrhoea is recommended. This will improve the culture diagnostics and provide additional gonococcal isolates for antimicrobial resistance surveillance.

## Background

The global spread of gonorrhoea and the ease with which *Neisseria gonorrhoeae* develops or acquires antibiotic resistance have led the World Health Organization (WHO) to describe gonorrhoea as an “urgent and major threat to public health” [1, 2]. Strategies to tackle the spread of infection and detect emerging antibiotic resistance as well as to monitor trends in antibiotic resistance are limited by both microbiological and clinical issues. An enhanced surveillance of gonococcal antibiotic resistance has been stated as imperative by the WHO [3].

Today the microbiological diagnosis of gonorrhoea is frequently based on nucleic acid amplification tests (NAATs), such as PCR. Culture of viable bacteria is necessary for phenotypic antibiotic susceptibility testing and, especially, culture is essential for the phenotypic or genetic detection of de novo antibiotic resistance mechanisms. Culture is also essential for detecting possible evolving new strains of gonococci that might escape NAAT detection due to mutations in target genes. Whole genome sequencing (WGS) for epidemiological studies also requires cultured isolates. The fastidious nature of *N. gonorrhoeae* is a well-known challenge to the success of culture [4].

The different clinical presentations of gonorrhoea, with varying *N. gonorrhoeae* loads at different mucosal surfaces have implications for the likelihood of success of culture. Urethritis often presents with acute copious discharge and high *N. gonorrhoeae* load, while infections in pharynx and anorectum are often asymptomatic and have lower bacterial loads [4, 5], which result in a lower culture rate especially for the pharyngeal infections. Asymptomatic extragenital infections are important to diagnose because they form a reservoir of untreated infection in society, unless patients take part in screening programs. Additionally, particularly infections in the pharynx are more difficult to treat and *N. gonorrhoeae* may also acquire antibiotic resistance genes from other commensals co-inhabiting this anatomical site [4, 6].

In Norway, a five-fold increase in the reported cases of gonorrhoea was observed during the decade (2006–2016) prior to the study [7]. A large proportion of these cases were diagnosed and treated at the Norwegian reference sexually transmitted infection (STI) clinic at Oslo University Hospital, located in central Oslo. The clinic has daily drop-in sessions, and had approximately 20 500 patient visits annually during the study period. Regular testing with a gonococcal PCR test is recommended for those with a high risk of STIs. In practice this offer is most often taken up by men who have sex with men (MSM). MSM are recommended to be routinely tested at three anatomical sites; pharynx, anorectum and urethra, whether they are symptomatic or asymptomatic. In 2014 the culture rate of gonococcal-positive PCR samples taken at the STI clinic was only 20% (1% in pharyngeal, 11% in anorectal, 39% in urethral and 26% in cervical samples). The Norwegian national antibiotic resistance surveillance figures for 2014 were based on only 255 (37%) available cultures from 682 *N. gonorrhoeae* PCR-positive gonorrhoea cases [7, 8].

To increase the number of *N. gonorrhoeae* isolates available for antimicrobial susceptibility testing, a prospective quality improvement study was conducted by the STI clinic and the Department of Microbiology at Oslo University Hospital from May 2016 - October 2017. The study aimed to improve the gonococcal culture rate of samples from different anatomical sites in symptomatic and asymptomatic patients. Bedside inoculation on gonococcal agar plates and incubation of samples at the STI clinic was compared to standard culture at the microbiology department.

## Materials and methods

### Inclusion and exclusion criteria

For a sample to be included, both case definition and technical criteria had to be fulfilled. Patients visiting the clinic with gonorrhoea fell naturally into two case definitions according to clinical presentation. Symptomatic patients were patients who referred themselves with symptoms and/or clinical signs, with microscopy findings suggestive of gonorrhoea and these patients were treated on the same day. The symptomatic site was cultured before the PCR result was known. Asymptomatic patients were patients without symptoms but with a recent *N. gonorrhoeae-*positive PCR result. The gonococcal PCR-positive site was cultured. Notably, during the study period no *N. gonorrhoeae-*negative PCR samples (n = 177) had a positive gonococcal culture.

The technical criteria for a sample to be included were two plates (standard and bedside culture) and a *N. gonorrhoeae-*positive PCR taken on the day of culture. To avoid dependency, only samples from the first visit during the study period were included.

Patients who received any antibiotic treatment in the 4 weeks before culture or developed symptoms between the screening PCR and the day of treatment, were excluded. Patients who had an initial screening PCR result from other laboratory than the Department of Microbiology at Oslo University Hospital, were also excluded. Finally, samples that proved PCR negative on the day of culture were excluded.

### Sampling methods

Opti-Swab 1 ml Liquid Amies Transport Medium with polyester HydraFlock swab (3506-H, Puritan) was used to sample all anatomical sites. The swab (not the liquid) was then immediately used to inoculate the agar plate for the culture at the clinic. All culture samples were taken before antibiotic treatment was administered.

Two swabs were taken from each sample site in random order.

Urethra: The external urethral meatus was swabbed. While urine is the material of choice for screening with PCR, no urine samples were cultured.

Pharynx: The posterior nasopharynx and the tonsillar arches were swabbed.

Anorectum: The swab was inserted 2–3 cm, moved back and forth/rotated.

Cervix: A vaginal speculum was used to provide a clear view of the cervix. The cervix was swabbed.

CLAT selective agar plates (Heart infusion agar (Difco), defibrinated horseblood, yeast autolysate supplement (OXOID SR 0105B) and LCAT selective supplement (Oxoid SR 0095B)) were used for both bedside and standard culture of samples. LCAT selective supplement contains colistin 3.0 mg, lincosamide 0.5 mg, amphotericin B 0.5 mg and trimethoprim 3.25 mg per 500 ml of medium, to inhibit growth of non-gonococcal bacterial and fungal species.

### Study structure

Patients with symptoms:

1) Swab for standard culture: The swab taken from the symptomatic site was placed directly in the transport medium, and stored immediately at 4 °C. Samples were transported twice daily in insulated boxes to the Department of Microbiology, a 10 min drive from the clinic. At the Department of Microbiology the liquid transport medium was vortexed and separated into two parts; one part was examined with a standard PCR for *N. gonorrhoeae*, *Chlamydia trachomatis* and *Mycoplasma genitalium*, and the other part was cultured on CLAT plates.

2) Swab for bedside culture: An incubator (set to 36 °C, 5% CO_2_-enriched atmosphere) was installed at the STI clinic. CLAT plates were prewarmed in the incubator. After sampling the symptomatic site, the swab was immediately placed and rolled on approximately 1/3 of the plate, then streaked out further with an inoculation loop. Plates were immediately incubated. The swab was then placed in its transport medium, stored at 4 °C and sent the same day for PCR.

Patients without symptoms:

1) Swab for standard culture: The swab from the *N. gonorrhoeae* PCR positive site was placed directly in the transport medium, stored at 4 °C and transported to the Department of Microbiology where the liquid was cultured according to the standard routine. As the PCR status of the sampling site was assumed to be positive from the earlier screening test, no PCR was performed from this swab.

2) Swab for bedside culture: This was performed in the same manner as for patients with symptoms, described above, including confirmatory PCR test.

CLAT plates from standard culture were inspected for growth on day 1 and day 2.

### Incubation and transportation of the bedside culture plates

Plates from Monday, Tuesday and Wednesday were incubated for 2 days at the STI clinic, and were first inspected for *N. gonorrhoeae* colonies at the Department of Microbiology on day 2. Samples plated on Thursday and Friday were all transported to the Department of Microbiology at the last transportation on Friday. Therefore Thursday plates were incubated for 1 day and Friday plates for a maximum of 4 h (0 days) at the clinic. At the Department of Microbiology the plates were inspected for *N. gonorrhoeae* colonies, the two day incubation period was completed and identification and antibiotic susceptibility testing were performed.

Transportation of plates was optimized by using special plastic pouches (Thermo Scientific Compact W-Zip Seal Pouches) containing a CO_2_ generator (Thermo Scientific Oxoid™ CO_2_Gen™ Compact Sachet). The sealed plastic pouches with plates were transported in a heated (36 °C) transport box (Portable Vaccine and Sample Carrier from LABCOLD) to the Department of Microbiology.

*N. gonorrhoeae* was species-verified using MALDI-TOF MS (Microflex LT MALDI-TOF with MALDI Biotyper software v.2.0, Bruker Daltonics, Bremen, Germany).

### PCR

PCR analysis was performed at the Department of Microbiology. A previously described duplex PCR targeting the *porA* pseudogene and *opa* genes was used [9]. If only one of the targets was positive, a confirmatory PCR (Fast-Track Diagnostics, Luxembourg) targeting another area of the *opa* genes and the *pilin* gene was applied. For a gonorrhoea diagnosis to be made, at least two different gonococcal targets had to be detected.

### Demographic and clinical characteristics

Microbiology results, relevant epidemiological and clinical information were retrospectively extracted from the electronic patient journal, and stored in an anonymised database.

### Statistical analysis

Sample size was calculated with a significance level α = 0.05, power 80%, one-sided superiority test. Culture results from 2014 were set as the baseline, and expected culture rates set at 50% for urethral, cervical and anorectal samples, and 10% for pharyngeal samples. For adequately powered analysis we required 252 urethral, 50 cervical, 17 anorectal and 79 pharyngeal samples. The null hypothesis that the intervention has no impact on the likelihood of obtaining a successful culture, was examined by McNemar’s test and the effect size of the intervention was examined by Odds Ratio (OR).

A linear logistic regression model with interaction was used to investigate differences in culture method success rate according to sample site. To facilitate the interpretation of significant differences between the two culture methods, estimated marginal means, i.e. the modelled culture rate, and their 95% confidence intervals were calculated and Wald tests were used for pairwise comparisons between methods within each sample site. An adjustment for 4 pairwise comparisons (performed on the log odds ratio scale) was performed using Holm method.

Data handling and analysis were performed in Statistical Package for the Social Sciences (SPSS) ‘IBM SPSS Statistics for Windows, version 26 (IBM Corp., Armonk, N.Y., USA), VassarStats Website for Statistical Computation (R. Lowry, http://vassarstats.net/) and R version 4.0.2. (R Core Team, http://www.R-project.org/).

### Ethics

The study was approved by the Privacy and Data Protection Officer at Oslo University Hospital, study reference: 2015–19428. Individual patient informed consent was not required.

## Results

### Study population

**Fig. 1:**
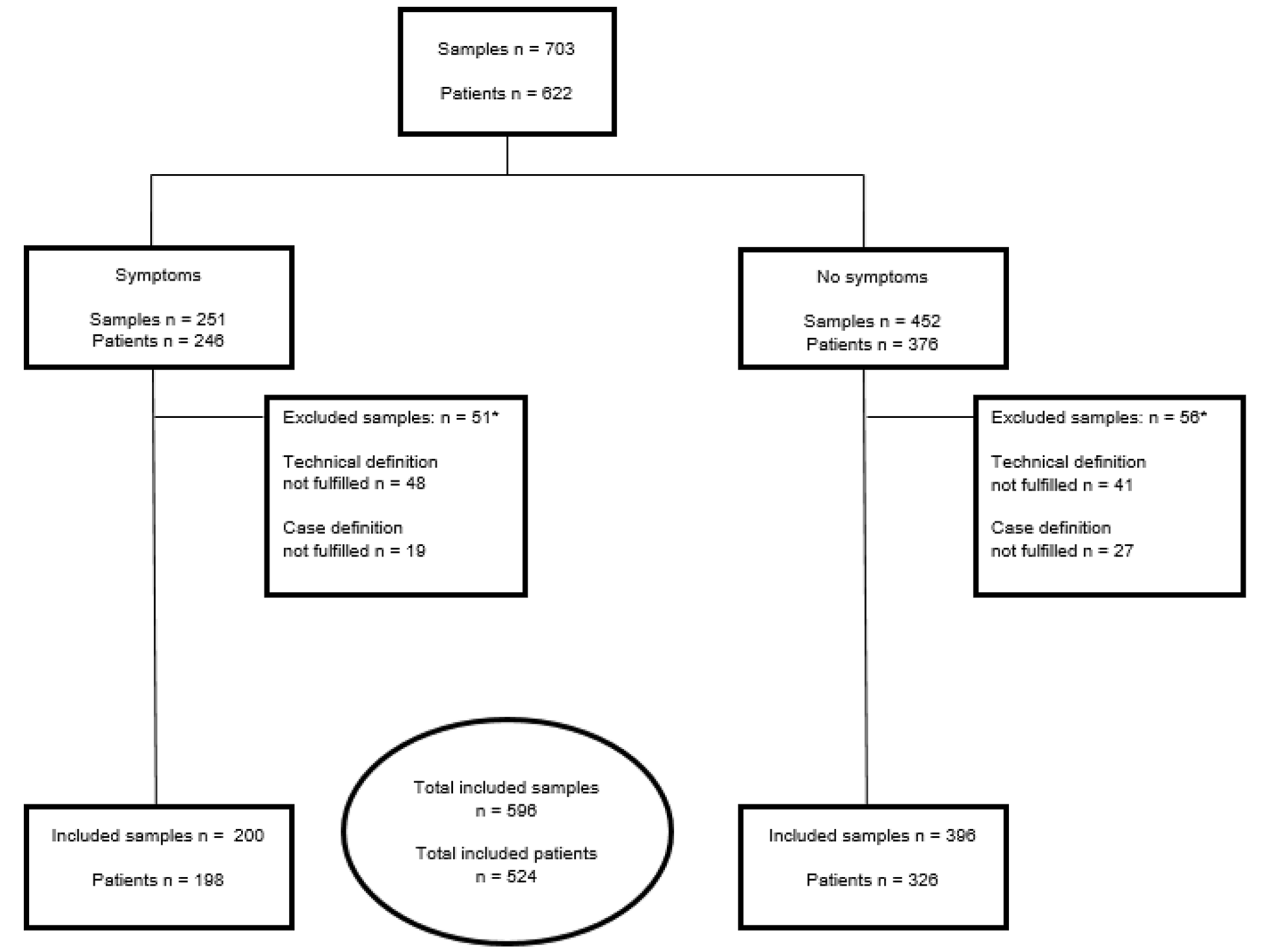
Flowchart showing *Neisseria gonorrhoeae* PCR-positive samples from patients from May 2016 - October 2017. *Some samples fail several inclusion criteria

Of the 703 eligible first visit samples (from 622 patients), 596 (85%) met the inclusion criteria. The 596 samples were from 524 individual patients: 200 samples were cultured from symptomatic sites and 396 from asymptomatic sites (Fig. 1). No patient had positive *N. gonorrhoeae* culture from both symptomatic and asymptomatic sites at their first visit.

Median age was 31 years for included patients with symptoms, and 30 years for patients without symptoms (range 16–82 years) (Table 1).

The majority (62%) of included patients were asymptomatic and these patients were detected by the routine screening test (PCR), 90% of these patients were MSM. In this asymptomatic MSM group, 285/294 (97%) had complete testing data (PCR from all three sample sites). None had urethral infection alone, 111 (39%) and 84 (29%) had pharyngeal and anorectal infection alone, respectively. Ninety (32%) were infected at more than one site (data not shown).

MSM also constituted the largest proportion of the symptomatic patients (56%), and 56% of these proved to be infected at more than one site (data not shown). Of the 97 MSM who presented with symptomatic urethritis, 73 were also PCR tested from both pharynx and anorectum. Of these 73, six (8.2%) were also found to have a PCR-positive gonococcal infection in the pharynx, 26 (36%) in anorectum, and 18 (25%) had a gonococcal infection at all three sites (data not shown).

The vast majority of men who have sex with women (MSW) and women who have sex with men (WSM) presented with symptoms.

**Table 1:** Characteristics of the included patients on their first visit in the study

	*Patient characteristics on the first visit*					
	Total		Symptoms		No symptoms	
	n	(%)	n	(%)	n	(%)
**Patients**	524	(100)	198	(100)	326	(100)
**Age** **(years)**						
15–24	97	(19)	30	(15)	67	(21)
25–34	240	(46)	94	(48)	146	(45)
35–44	121	(23)	44	(22)	77	(24)
> 45	66	(13)	30	(15)	36	(11)
**Sexual behaviour**						
MSM	405	(77)	111	(56)	294	(90)
MSW	67	(13)	58	(29)	9	(2.8)
WSM	27	(5.2)	19	(9.6)	8	(2.5)
MSW/M	25	(4.8)	10	(5.1)	15	(4.6)

MSM, men who have sex with men; MSW, men who have sex with women; WSM, women who have sex with men; MSW/M, men who have sex with women and men.

### Culture rate results

The *N. gonorrhoeae-*positive PCR samples were divided into four groups based on the combination of bedside and standard culture results (Table 2):

1) Culture negative (n = 237, 40%).

2) Bedside culture positive, standard culture negative (n = 116, 20%).

3) Bedside culture negative, standard culture positive (n = 21, 3.5%).

4) Bedside culture positive, standard culture positive (n = 222, 37%).

The culture rate was significantly higher using bedside culture (57%) compared to standard culture (41%, p < 0.05). The total culture rates (bedside plus standard) in the different anatomical sites were as follows: pharynx 28%, anorectum 64%, urethra 93% and cervix 70%. Significant improvement was seen for bedside culture of urethra and pharynx (p < 0.0125 with Bonferroni correction).

**Table 2a and 2b:** Bedside *v.* standard culture from PCR-positive sample sites in symptomatic and asymptomatic patients

	*Symptomatic patients* *Bedside / Standard culture result* n (% per sample site)				
2a)	Anorectum	Pharynx	Urethra*	Cervix	All sample sites
Negative/negative	5 (33)	1 (100)	9 (5.4)	3 (17)	18 (9.0)
Positive/negative	0	0	41 (25)	6 (33)	47 (24)
Negative/positive	0	0	2 (1.2)	0	2 (1.0)
Positive/positive	10 (67)	0	114 (69)	9 (50)	133 (67)
Total	15	1	166	18	200
	***Asymptomatic patients*** ***Bedside / Standard culture result*** ***n (% per sample site)***				
2b)	Anorectum	Pharynx*	Urethra	Cervix	All sample sites
Negative/negative	61 (36)	151 (72)	3 (25)	4 (80)	219 (55)
Positive/negative	24 (14)	43 (21)	2 (17)	0	69 (17)
Negative/positive	14 (8.2)	4 (1.9)	0	1 (20)	19 (4.8)
Positive/positive	71 (42)	11 (5.3)	7 (58)	0	89 (23)
Total	170	209	12	5	396

*p < 0.05, significant difference in culture rate between bedside and standard culture (McNemar’s test).

From symptomatic patients, 200 samples were PCR-positive for *N. gonorrhoeae* (Table 2a). A total of 182 samples were *N. gonorrhoeae* culture positive, giving a culture rate of 91%. Samples were mainly from urethra, which achieved a culture rate of 95%. Culture rate from urethra was significantly higher using bedside *v.* standard culture (p < 0.05). For the cervix, pharynx and anorectum numbers were too small to perform a McNemar’s test.

From asymptomatic patients, 396 samples were PCR-positive for *N. gonorrhoeae* (Table 2b) and culture rate was 45% (177/396). Samples were mainly from the pharynx and anorectum which achieved culture rates of 28% and 64% respectively. Pharynx had a significantly higher culture rate using bedside *v.* standard culture (p < 0.05). Anorectal samples did not show a significant difference. There were too few samples from urethra and cervix to perform a McNemar’s test.

**Fig. 2:**
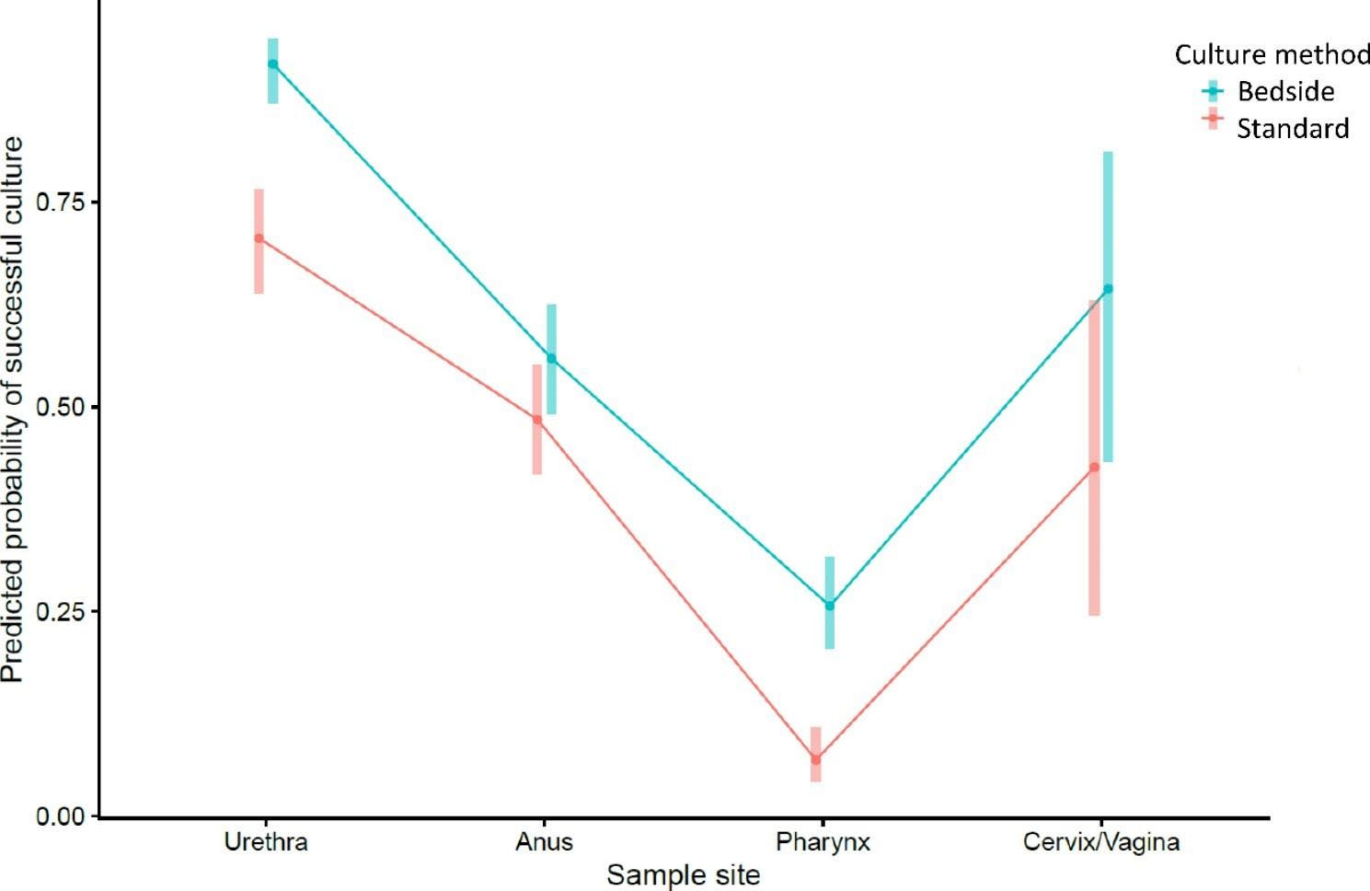
Predicted probability for each culture method at each sample site with corresponding 95% confidence interval

To further investigate the probability of obtaining a successful culture, a linear logistic regression model was used. Predictor variables included culture method, sample site, and the interaction term between sample site and culture method for the modelling (Fig. 2).

We computed post-estimation contrasts (i.e. ORs) to determine the total effect size of the bedside culture *v.* standard culture across sample sites (Table 3). An OR of 1 would indicate no difference in the culture routines, OR > 1 indicates that bedside culture is superior.

**Table 3:** Odds ratios for Bedside culture *v.* Standard culture methods

Sample Site	Odds Ratio	Standard Error	Confidence Interval	P value
Urethra	4.73	1.44	(1.91, 7.55)	< 0.0001*
Anorectum	1.35	0.26	(0.84, 1.86)	0.1219
Pharynx	4.70	1.40	(1.94, 7.43)	< 0.0001*
Cervix	2.44	1.48	(-0.46, 5.34)	0.1422

Significance of the effect size/OR calculated by Wald test (*p < 0.05).

### Incubation time at the STI clinic and seasonality

Culture rates of samples incubated at the STI clinic for 2 days were 54% (95%CI: 49–59%), 1 day 67% (95%CI: 57–75%) and 0 days 58% (95%CI: 49–67%). There was no significant difference in culture success rates according to length of incubation at the STI clinic (Chi-square p > 0.05). No seasonal effect on likelihood of culture success was detected.

### Outcome of the study on patient level

Without the bedside inoculation and incubation at the STI clinic, 108/524 patients (with 116 samples) would have had no culture positive sample. Of these patients, 46 were symptomatic and 62 were asymptomatic.

## Discussion

The culture rate was significantly higher using bedside culture (57%) compared to standard culture (41%, p < 0.05). The linear logistic regression model illustrates the superiority of the bedside culture for all sample sites, but statistical significance is only attained for urethra and pharynx, where the confidence intervals do not overlap (Fig. 2). The ORs shown in Table 3 underscore these results, with a highly significant effect size of the bedside culture intervention for urethra and pharynx. Based on these results, bedside culture was implemented in the clinic routine in 2017. The 108 patients who would otherwise not have received an antibiotic resistance report make up a considerable proportion of the study group (21%).

An increased focus on quality-assured gonococcal culture during the study at the Department of Microbiology could plausibly explain the increase in their culture rate using standard culture, from 20% to 2014 to 41% during this study. In practice, it did not make any difference if the inoculated agar plates were incubated for the full 48 h at the clinic or transported within a few hours after plating. This, and the lack of seasonal effects despite Norwegian seasonal temperature variation, indicates that the transportation system provided adequate continuous protection of atmosphere and temperature. This could be borne in mind in less-resourced settings that do not allow for installation and maintenance of an incubator.

Symptomatic urethra samples had the best culture rate. Bedside culture at the STI clinic gave a success rate of 93%, while the Department of Microbiology`s culture rate using standard culture was 70% (39% in 2014). The performance using bedside culture at the STI clinic is the same as Radebe et al. found for urethral samples (also 93% culture rate) when they performed direct cultures from 100 patients with symptomatic urethritis [10].

The total culture rate for pharyngeal samples improved considerably from 2014 (from 1 to 28%), with significant improvement seen through bedside culture (26% *v.* 7.1% using standard culture). Our result is similar to other published studies [11, 12].

As far as we know, the highest reported *N. gonorrhoeae* culture rate from pharynx is about 40% [13–15]. Several factors may explain the poorer culture results from the pharynx. Bissessor et al. [5] suggested asymptomatic pharyngeal infections have a lower *N. gonorrhoeae* load compared to in anorectal infections, and that the *N. gonorrhoeae* load in the pharynx is higher in those culture positive compared to those who are culture negative. Chow et al. also indicated this to be true, though their results did not reach statistical significance [16]. Swabbing technique is important. Mitchell et al. [17] concluded that swabbing of both the tonsils and oropharynx with reasonable pressure of the oropharynx improved culture positivity.

Samples from the anorectum were relatively difficult to culture compared to urogenital samples, in common with other studies [18, 19]. The effect size of bedside culture *v.* standard culture for anorectal samples is smaller than for the other anatomical sites (Table 3). This may be due to the 14 anorectal samples from asymptomatic patients that were culture positive only with standard culture. This seems biologically unlikely, and may represent a technical issue. The difference in inoculation technique between the laboratory and the STI clinic may explain this result. Inoculation by rolling the swab on the surface of the plate at the clinic, may have resulted in an overly thick bacterial growth. Gram-negative anorectal commensal flora was frequently observed to dominate the plates from the clinic. This in turn may have resulted in an overgrowth of the gonococci resulting in reports of no gonococcal growth. This also indicates that the plates were not sufficiently selective.

The total culture rate for anorectum from the relatively small group of patients with symptoms was 67% (10/15) and for patients without symptoms 64% (109/170). This is in contrast to Bissessor et al. who found that symptomatic anorectal infections had a higher bacterial load and greater chance of positive culture, compared to asymptomatic infections [5].

Despite all efforts to optimize culture, 40% of all *N. gonorrhoeae* PCR-positive samples were culture negative. Most (92%) of these samples were from asymptomatic patients. 64% were pharyngeal samples, so low bacterial load [4–6, 15] may be an explanation. Another potential explanation may be that the non-growing isolates were susceptible for any of the antimicrobials added to the CLAT-plate (colistin, lincosamide and/or trimethoprim) [20].

Other studies have divided their patients into groups based on sexual behaviour and concluded that being a MSM is a risk factor for negative culture [18, 19]. The majority of our included patients were MSM (77%). Regular screening in this group detects asymptomatic anorectal and pharyngeal infections. We find it more likely that it is the characteristics of the sample site that impact on the likelihood of a positive culture, not the sexual behaviour per se.

### Should we recommend culturing several sample sites on symptomatic MSM?

The most common presentation of symptomatic gonorrhoea is urethritis, and the clinical suspicion is easily confirmed by microscopy of urethral discharge during the consultation. Culture must be taken before antibiotics are administered. It may be tempting to only culture from easily available urethral discharge, but different antimicrobial resistance patterns from different anatomical sites in the same patient have been observed in a Norwegian dataset [21]. When resources permit, should we attempt to culture all sites in high risk patients, before antibiotic treatment, before all the confirmatory PCR results are known?

In the present study, 73 MSM with symptomatic urethritis had followed the recommended testing protocol of the three localisations urethra, anorectum and pharynx. Of these only 23 (32%) had urethritis alone. If culturing blind, the combined probability of finding an asymptomatic anorectal infection that results in a successful culture would be 39%. The cost benefit of culturing blind depends on the health care setting and resource allocation.

### Limitations

Urethral and cervical samples were underpowered. The initial power of the study calculations were made on the basis of culture rates per sample sites from 2014. Unfortunately it was not recorded whether 2014 cases were ‘symptomatic’ or not. The study was extended in an attempt to reach the required numbers, but the natural differences in clinical presentations made it impossible to collect sufficient numbers for all sample sites with the time and resources available. For symptomatic urethral samples the effect size and the statistical significance (p < 0.000001) were so strong, that we were not concerned that we were risking a type II error. However, for cervical samples the small sample size hindered statistical analysis. Though the effect size (OR) was favourable, few cases and overlapping 95%CI of the probability of getting a positive culture made it difficult to reach a definitive conclusion about the effect of the intervention. The study was not optimally designed for female patients. The women in our study generally presented first to their general practitioner where they often received antibiotics for presumed chlamydia or bacterial vaginosis, prior to the gonococcal PCR result being known. Therefore, women often fell between the two case definitions and were disproportionately excluded.

## Conclusions

Bedside culture is a relatively simple and cheap intervention and it resulted in a significant improvement in *N. gonorrhoeae* culture rates from urethral samples in symptomatic patients and for pharyngeal samples in asymptomatic patients. The effect size (OR) was greater than 1 for all sample sites, indicating that the bedside culture was superior. We recommend bedside inoculation and incubation of all samples from patients with gonorrhoea at the STI clinic to optimise culture rates of *N. gonorrhoeae.*

The high level of gonococcal infections in multiple anatomical sites in the MSM group, supports the recommendations to test with PCR across all three main sites (urogenital, anorectal and pharyngeal) in both symptomatic and asymptomatic patients. When MSM present with symptomatic urethritis, it may be worth culturing additional localisations (anorectum and pharynx) before confirmatory PCR results are known.

Despite our best efforts to optimise the conditions for *N. gonorrhoeae* culture, 40% of *N. gonorrhoeae*-positive PCR samples remained culture negative. Most (92%) of these samples were from asymptomatic patients, 69% were pharyngeal samples. Further studies should focus on optimizing culture rate from pharynx.

## Data Availability

The datasets generated during the current study are available from the corresponding authors on reasonable request.
